# Supplementary material for: Pharmacological enrichment of polygenic risk for precision medicine in complex disorders
Source: Sci Rep. 2020 Jan 21;10:879. doi: 10.1038/s41598-020-57795-0 (PMC6972917; doi:10.1038/s41598-020-57795-0)
Supplement: Supplementary file 1 — Supplementary Information. [file 41598_2020_57795_MOESM1_ESM.pdf]

Supplementary Materials – Pharmacological enrichment of polygenic risk for precision medicine in complex disorders

William R. Reay[1,2], Joshua R. Atkins[1,2], Vaughan J. Carr[3,4,5], Melissa J. Green[3,4], Murray J. Cairns[1,2] *

[1]School of Biomedical Sciences and Pharmacy, The University of Newcastle, Callaghan, NSW, Australia

[2]Centre for Brain and Mental Health Research, Hunter Medical Research Institute, Newcastle, NSW, Australia

[3]School of Psychiatry, University of New South Wales, Randwick, NSW, Australia

[4]Neuroscience Research Australia, Sydney, NSW, Australia

[5]Department of Psychiatry, Monash University, Melbourne, VIC, Australia

Supplementary Fig 1. Methodology for identifying pharmacologically-relevant pathways enriched with GWAS risk variants.

Supplementary Fig 2. Tissue specific expression of genes contained within candidate PES pathways derived from schizophrenia GWAS

Supplementary Fig. 3. Distribution of schizophrenia and healthy control patients with multiple elevated *pharmagenic enrichment scores*.

Supplementary Table 1. Characteristics of the candidate PES profiles.

Supplementary Table 2. Overrepresentation of genes within candidate PES pathways in the GWAS catalogue traits with relevance to psychiatry after multiple testing correction.

Supplementary Table 3. Highest confidence drug interaction between of a member of each pathway enriched with common polygenic risk for schizophrenia.

Supplementary Table 4. Enriched drug targets for each *pharmagenic enrichment score* with at least three interacting genes after multiple testing correction (FDR < 0.05)

Supplementary Table 5. Geometric characteristics of the Gaussian models used for parameterisations of the within-group covariance matrix.

Supplementary Table 7. Characteristics of the ASRB cohort analysed using the PES methodology**.**

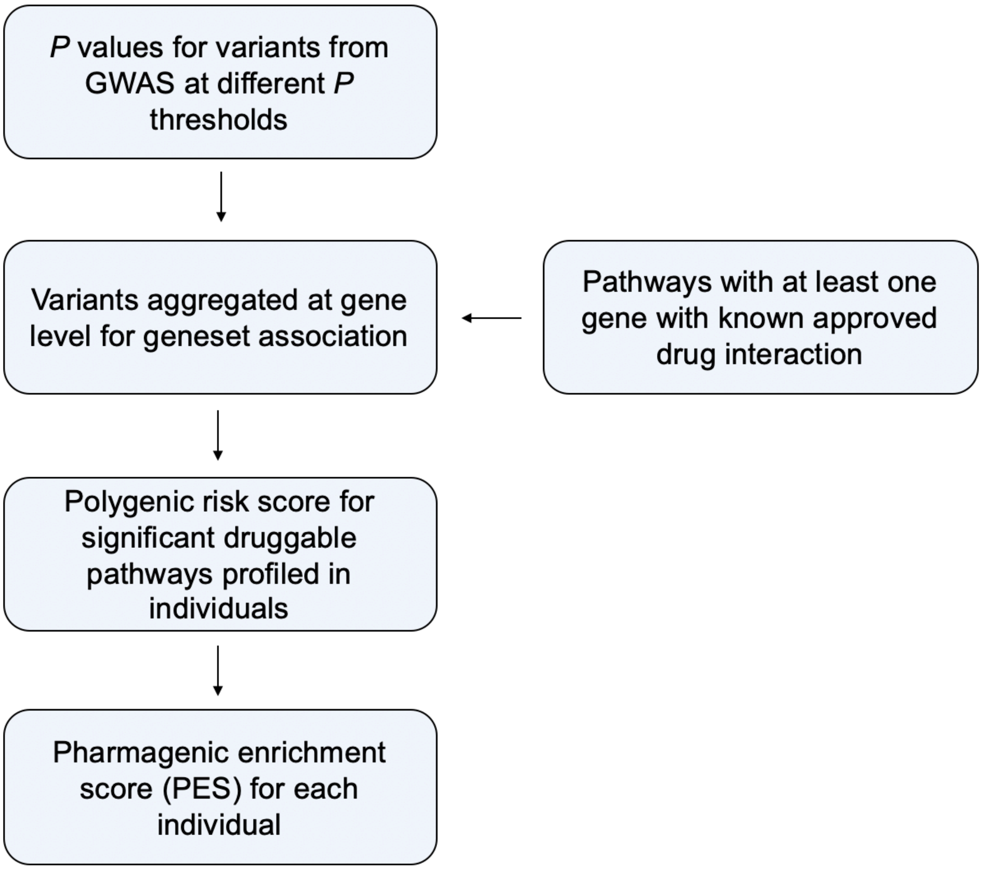

Supplementary Fig 1. Methodology for identifying pharmacologically-relevant pathways enriched with GWAS risk variants. The combined effect of variants (*P* values) from a genome wide association study (GWAS) is tested at level of genes. Different *P* value thresholds (P_T) are used to filter variants for input to capture biological signals only present at varying levels of polygenicity. Using a regression approach implemented by the MAGMA algorithim, geneset association is undertaken at each P_T and pathways are then filtered based on likelihood of interaction with an approved drug. Polygenic risk score (PRS) is constructed for each significant pathway uncovered via the pipeline to formulate a *pharmagenic enrichment score (PES)*. This PES can then be profiled in individuals to reveal participants with elevated PES, which in turn may be relevant to treatment formulation.

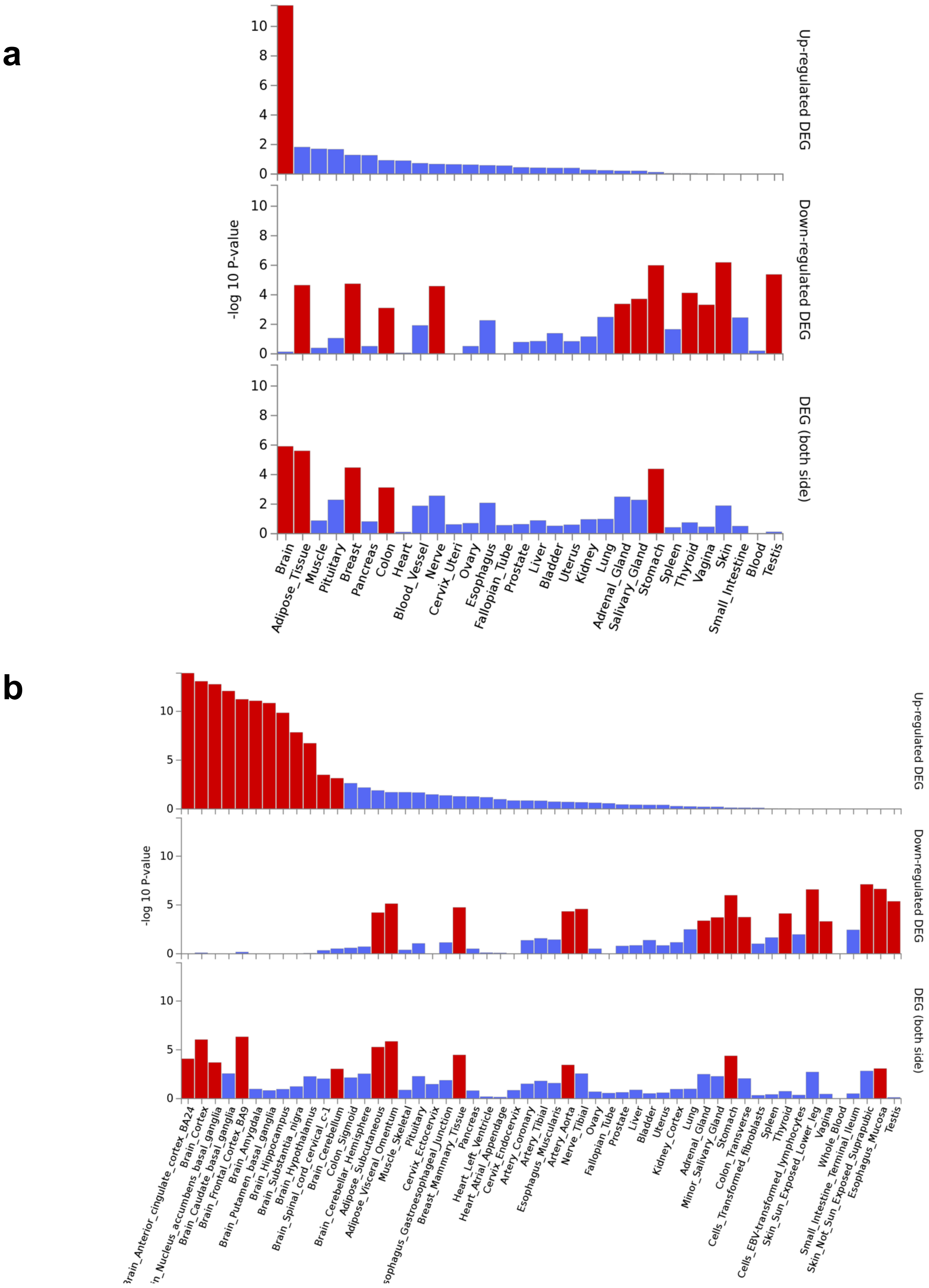
a
b
-log 10 P-value
Up-regulated DEG
Down-regulated DEG
DEG (both side)
0
2
4
6
8
10
5
Brain
Adipose_Tissue
Muscle
Pituitary
Breast
Pancreas
Colon
Heart
Blood_Vessel
Nerve
Cervix_Uteri
Ovary
Esophagus
Fallopian_Tube
Prostate
Liver
Bladder
Uterus
Kidney
Lung
Adrenal_Gland
Salivary_Gland
Stomach
Spleen
Thyroid
Vagina
Skin
Small_Intestine
Blood
Testis
Brain_Anterior_cingulate_cortex_BA24
Brain_Cortex
in_Nucleus_accumbens_basal_ganglia
Brain_Caudate_basal_ganglia
Brain_Frontal_Cortex_BA9
Brain_Amygdala
Brain_Putamen_basal_ganglia
Brain_Hippocampus
Brain_Substantia_nigra
Brain_Hypothalamus
Brain_Spinal_cord_cervical_c-1
Brain_Cerebellum
Colon_Sigmoid
Brain_Cerebellar_Hemisphere
Adipose_Subcutaneous
Adipose_Visceral_Omentum
Muscle_Skeletal
Pituitary
Cervix_Ectocervix
sophagus_Gastroesophageal_Junction
Breast_Mammary_Tissue
Pancreas
Heart_Left_Ventricle
Heart_Atrial_Appendage
Cervix_Endocervix
Artery_Coronary
Artery_Tibial
Esophagus_Muscularis
Artery_Aorta
Nerve_Tibial
Ovary
Fallopian_Tube
Prostate
Liver
Bladder
Uterus
Kidney_Cortex
Lung
Adrenal_Gland
Minor_Salivary_Gland
Stomach
Colon_Transverse
Cells_Transformed_fibroblasts
Spleen
Thyroid
Cells_EBV-transformed_lymphocytes
Skin_Sun_Exposed_Lower_leg
Vagina
Whole_Blood
Small_Intestine_Terminal_Ileum
Skin_Not_Sun_Exposed_Suprapubic
Esophagus_Mucosa
Testis

Supplementary Fig 2. Tissue specific expression of genes contained within candidate PES pathways derived from schizophrenia GWAS. Expression per tissue for genes which comprise these pathways was compared to the rest of the protein coding genome, with the -$\log_{10}$(*P*-value) reported for each test after the application of multiple testing correction (red bars indicating tissues which survive correction). Tissue specific expression was performed to assess up-regulation, downregulation and a two-sided test of differential expression. (**a**) GTEx v7 30 tissue types. (**b**) GTEx v7 53 tissue types.

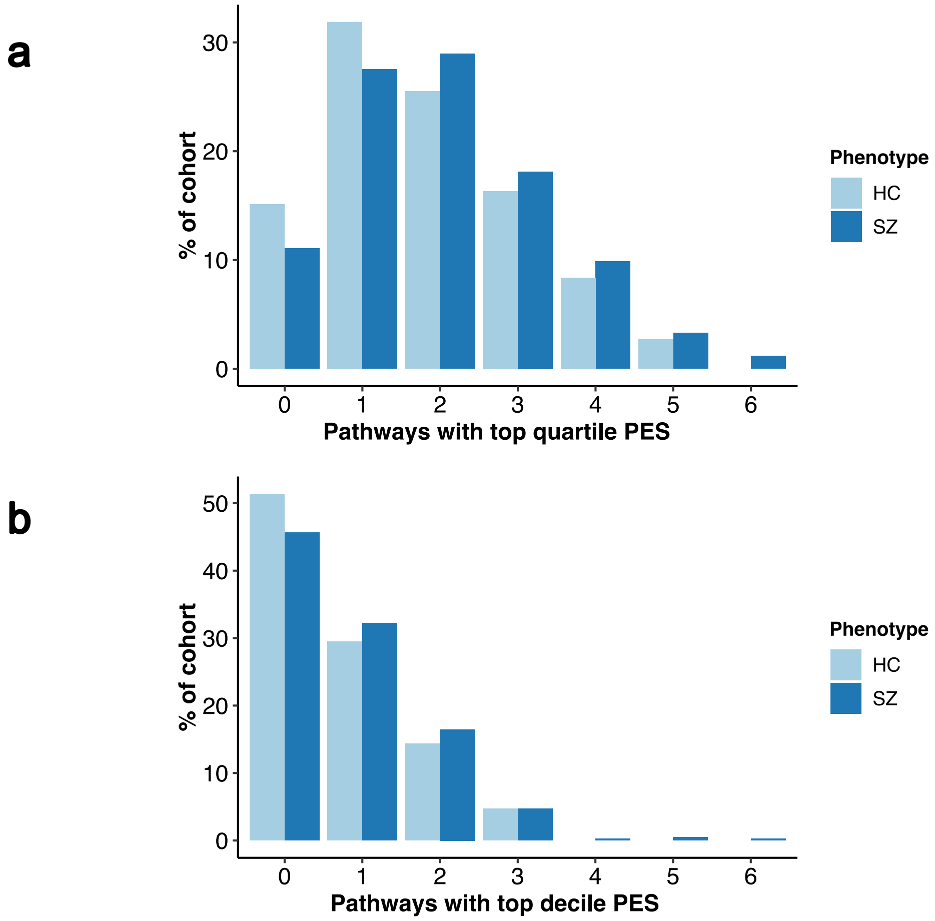

Supplementary Fig 3. Distribution of schizophrenia and healthy control patients with multiple elevated *pharmagenic enrichment scores*. PES in each pathway in the top quartile (**a**) or decile (**b**) of the ASRB cohort are classified as high and the number of scores over this threshold counted in each individual, represented here as a percentage of each phenotype cohort (that is, SZ or HC). The highest number of top quartile or decile PES scores in an individual is six (only schizophrenia patients). SZ = schizophrenia, HC = healthy controls.

Pathway	P threshold (P_T)	NGenes	NSNPs
NOS1 pathway	All SNPs	22	55
Regulation of insulin secretion	$P < 0.5$	88	337
CRMPs in Sema3A signalling	$P < 0.5$	15	94
GABA synthesis, release, reuptake and degradation	$P < 0.5$	23	183
One carbon pool by folate	$P < 0.05$	17	14
Hedgehog signalling	$P < 0.05$	36	46
HIF-2 pathway	$P < 0.005$	35	9
Acetylcholine binding and downstream events	$P < 0.005$	15	6

Supplementary Table 1. Characteristics of the candidate PES profiles. The pathways which were used to construct the PES, with the number of genes in the pathway listed (NGenes). The *P*-value threshold for inclusion of SNPs and the number of SNPs (NSNPs) mapped to the pathway genes which remained after clumping during the score calculation (NSNPs) are also detailed.

Phenotype	Overlap	Adjusted *P*-value
Nicotine dependence	7	4.28×10^{-8}
Smoking behaviour	5	8.95×10^{-5}
Schizophrenia	18	0.0023
Excessive daytime sleepiness	2	0.017
Post-traumatic stress disorder	2	0.021
Hippocampal volume	2	0.024
PGC cross disorder	3	0.033
Cognitive performance	4	0.034
White matter hyperintensity burden	2	0.035
Night sleep phenotypes	10	0.036
Social communication problems	2	0.044
Cerebral amyloid deposition in APOEε4 non-carriers (PET imaging)	2	0.044

Supplementary Table 2. Overrepresentation of genes within candidate PES pathways in the GWAS catalogue traits with relevance to psychiatry after multiple testing correction. Overlap column pertains to genes common to the PES geneset and the trait geneset

Pathway	Drug*	ATC Code	ATC Code Level 4
NOS1	Glycine	B05CX03	Other irrigating solutions
GABA	Baclofen	M03BX01	Other centrally acting agents#
CRMPs Sema3A	Dasatinib	L01XE06	Protein kinase inhibitors
HIF-2	Sunitinib	L01XE04	Protein kinase inhibitors
Acetylcholine	Varenicline	N07BA03	Drugs used in nicotine dependence
Hedgehog	Tacrine	N06DA01	Anticholinesterases
Folate	Trifluridine	S01AD02	Antivirals
Insulin	Exenatide	A10BJ01	Glucagon-like-peptide-1 (GLP-1) analogue

Supplementary Table 3. Highest confidence drug interaction between of a member of each pathway enriched with common polygenic risk for schizophrenia. Drugs selected by highest confidence interaction score with a gene (classified as T_{Clin}) in each of the pathways by DGidb v3.02

PES PATHWAY	DRUGBANK ID	DRUG NAME
NOS1	DB06741	Gavestinel
NOS1	DB04896	Milnacipran
NOS1	DB00289	Atomoxetine
NOS1	DB06738	Ketobemidone
NOS1	DB00996	Gabapentin
NOS1	DB01174	Phenobarbital
NOS1	DB01520	Tenocyclidine
NOS1	DB00454	Pethidine
NOS1	DB00418	Secobarbital
NOS1	DB02868	3"-(Beta-Chloroethyl)-2",4"-Dioxo-3, 5"-Spiro-Oxazolidino-4-Deacetoxy-Vinblastine
NOS1	DB06151	Acetylcysteine
NOS1	DB00659	Acamprosate
NOS1	DB00312	Pentobarbital
NOS1	DB01429	Aprindine
NOS1	DB03977	N-Trimethyllysine
NOS1	DB04825	Prenylamine
NOS1	DB08039	(3Z)-N,N-DIMETHYL-2-OXO-3-(4,5,6,7-TETRAHYDRO-1H-INDOL-2-YLMETHYLIDENE)-2,3-DIHYDRO-1H-INDOLE-5-SULFONAMIDE
NOS1	DB01708	Dehydroepiandrosterone
NOS1	DB00527	Cinchocaine
NOS1	DB00623	Fluphenazine
NOS1	DB00850	Perphenazine
NOS1	DB04513	N-(6-Aminohexyl)-5-Chloro-1-Naphthalenesulfonamide
NOS1	DB01043	Memantine
NOS1	DB01100	Pimozide
NOS1	DB03900	2-Methyl-2-Propanol
NOS1	DB04841	Flunarizine
NOS1	DB08231	Myristic acid
NOS1	DB00831	Trifluoperazine
NOS1	DB00836	Loperamide
NOS1	DB00925	Phenoxybenzamine
NOS1	DB01115	Nifedipine
NOS1	DB01065	Melatonin
NOS1	DB01244	Bepridil
NOS1	DB01373	Calcium
NOS1	DB01069	Promethazine
NOS1	DB00142	L-Glutamic Acid
NOS1	DB01023	Felodipine
NOS1	DB00949	Felbamate

NOS1	DB02527	Cyclic Adenosine Monophosphate
NOS1	DB00622	Nicardipine
NOS1	DB00753	Isoflurane
NOS1	DB00477	Chlorpromazine
NOS1	DB01173	Orphenadrine
NOS1	DB00163	Vitamin E
GABA	DB00186	Lorazepam
GABA	DB00189	Ethchlorvynol
GABA	DB00228	Enflurane
GABA	DB00231	Temazepam
GABA	DB00237	Butabarbital
GABA	DB00241	Butalbital
GABA	DB00273	Topiramate
GABA	DB00292	Etomidate
GABA	DB00306	Talbutal
GABA	DB00312	Pentobarbital
GABA	DB00349	Clobazam
GABA	DB00371	Meprobamate
GABA	DB00402	Eszopiclone
GABA	DB00404	Alprazolam
GABA	DB00463	Metharbital
GABA	DB00475	Chlordiazepoxide
GABA	DB00546	Adinazolam
GABA	DB00628	Clorazepate
GABA	DB00659	Acamprosate
GABA	DB00683	Midazolam
GABA	DB00690	Flurazepam
GABA	DB00753	Isoflurane
GABA	DB00794	Primidone
GABA	DB00801	Halazepam
GABA	DB00818	Propofol
GABA	DB00829	Diazepam
GABA	DB00842	Oxazepam
GABA	DB00897	Triazolam
GABA	DB01028	Methoxyflurane
GABA	DB01049	Ergoloid
GABA	DB01068	Clonazepam
GABA	DB01107	Methyprylon
GABA	DB01159	Halothane
GABA	DB01189	Desflurane
GABA	DB01205	Flumazenil
GABA	DB01215	Estazolam
GABA	DB01236	Sevoflurane
GABA	DB01437	Glutethimide
GABA	DB01558	Bromazepam

GABA	DB01559	Clotiazepam
GABA	DB01567	Fludiazepam
GABA	DB01588	Prazepam
GABA	DB01589	Quazepam
GABA	DB01594	Cinolazepam
GABA	DB01595	Nitrazepam
GABA	DB01708	Dehydroepiandrosterone
GABA	DB11582	Thiocolchicoside
GABA	DB00543	Amoxapine
GABA	DB00334	Olanzapine
GABA	DB05087	Ganaxolone
GABA	DB00898	Ethanol
GABA	DB00849	Methylphenobarbital
GABA	DB01351	Amobarbital
GABA	DB01352	Aprobarbital
GABA	DB01353	Butethal
GABA	DB01354	Heptabarbital
GABA	DB01355	Hexobarbital
GABA	DB01483	Barbital
GABA	DB01496	Barbituric
GABA	DB00599	Thiopental
GABA	DB01544	Flunitrazepam
GABA	DB00418	Secobarbital
GABA	DB01198	Zopiclone
GABA	DB00425	Zolpidem
GABA	DB01381	Ginkgo
GABA	DB01587	Ketazolam
GABA	DB00466	Picrotoxin
GABA	DB01346	Quinidine
Insulin	DB00720	Clodronate
Insulin	DB00661	Verapamil
HIF-2	DB00126	Vitamin C
Acetylcholine	DB00184	Nicotine
Acetylcholine	DB00674	Galantamine
Acetylcholine	DB00898	Ethanol
Acetylcholine	DB05740	RPI-78M
Acetylcholine	DB01273	Varenicline
Acetylcholine	DB09028	Cytisine
Acetylcholine	DB00514	Dextromethorphan
Acetylcholine	DB00849	Methylphenobarbital
Acetylcholine	DB01351	Amobarbital
Acetylcholine	DB01352	Aprobarbital
Acetylcholine	DB01353	Butethal
Acetylcholine	DB01354	Heptabarbital
Acetylcholine	DB01355	Hexobarbital
Acetylcholine	DB01483	Barbital

Acetylcholine	DB01496	Barbituric acid
Acetylcholine	DB00599	Thiopental
Acetylcholine	DB01174	Phenobarbital
Acetylcholine	DB01090	Pentolinium
Acetylcholine	DB01227	Levomethadyl
Acetylcholine	DB00418	Secobarbital
Acetylcholine	DB00237	Butabarbital
Acetylcholine	DB00241	Butalbital
Acetylcholine	DB00306	Talbutal
Acetylcholine	DB00463	Metharbital
Acetylcholine	DB00794	Primidone
Acetylcholine	DB00312	Pentobarbital
Folate	DB00116	Tetrahydrofolic acid
Folate	DB00642	Pemetrexed

Supplementary Table 4. Enriched targets for each pharmagenic enrichment score pathway with at least three interacting (overlapping) genes after multiple testing correction (FDR adjusted $P < 0.05$).

**Model abbreviation***	**Distribution**	**Volume**	**Shape**	**Orientation**
EEI	Spherical	Equal	Equal	-
VII	Spherical	Variable	Equal	-
EEI	Diagonal	Equal	Equal	Coordinate axes
VEI	Diagonal	Variable	Equal	Coordinate axes
EVI	Diagonal	Equal	Variable	Coordinate axes
VVI	Diagonal	Variable	Variable	Coordinate axes
EEE	Ellipsoidal	Equal	Equal	Equal
EVE	Ellipsoidal	Equal	Variable	Equal
VEE	Ellipsoidal	Variable	Equal	Equal
VVE	Ellipsoidal	Variable	Variable	Equal
EEV	Ellipsoidal	Equal	Equal	Variable
VEV	Ellipsoidal	Variable	Equal	Variable
EVV	Ellipsoidal	Equal	Variable	Variable
VVV	Ellipsoidal	Variable	Variable	Variable

Supplementary Table 5. Geometric characteristics of the Gaussian models used for parameterisations of the within-group covariance matrix. Gaussian models described as implemented in the mclust package.

	Control	Case
Total	251	425
Males	110	283
Females	141	142
Mean Age (s.d.)	39.50 (13.40)	39.88 (10.92)
Mean Onset Age (s.d.)	N/A	23.79 (6.89)
Mean GAF score (s.d.)	84.13(8.81)	53.56 (13.43)

Supplementary Table 7. Characteristics of the ASRB cohort to which the PES pipeline was applied to – cases refer to subjects diagnosed with schizophrenia. GAF refers to the global assessment of functioning scale, with a lower score indicating greater symptom severity for the disorder.
